# Cellulose and Lignin Nano-Scale Consolidants for Waterlogged Archaeological Wood

**DOI:** 10.3389/fchem.2020.00032

**Published:** 2020-01-29

**Authors:** Federica Antonelli, Giulia Galotta, Giancarlo Sidoti, Florian Zikeli, Rossella Nisi, Barbara Davidde Petriaggi, Manuela Romagnoli

**Affiliations:** ^1^Department for Innovation in Biological, Agro-Food and Forestry Systems (DIBAF), Tuscia University, Viterbo, Italy; ^2^Biology Laboratory, Istituto Superiore per la Conservazione e il Restauro (ISCR), Rome, Italy; ^3^Testing Materials Laboratory, Istituto Superiore per la Conservazione e il Restauro (ISCR), Rome, Italy; ^4^BioFaber srl, Mesagne, Italy; ^5^Underwater Archaeological Operations Unit, Istituto Superiore per la Conservazione ed il Restauro (ISCR), Rome, Italy

**Keywords:** lignin nanoparticles (LNP), bacterial cellulose (BC), cellulose nanocrystals (CNC), equilibrium moisture content (EMC), anti-shrink efficiency (ASE), SEM, cultural heritage

## Abstract

Waterlogged archaeological wood comes from submerged archaeological sites (in lake, sea, river, or wetland) or from land waterlogged sites. Even if the wooden object seems to have maintained the original size and shape, the wood is more or less severely decayed because of chemical and biological factors which modify the normal ratio of cellulose and lignin in the cell wall. Drying procedures are necessary for the musealization but potentially cause severe shrinkages and collapses. The conservation practices focus not only on removing water from wood but also on substituting it with materials able to consolidate the degraded wood cell walls like polymers (e.g., PEG), sugars (e.g., lactitol), or resins (e.g., Kauramin). In the present work three different nano-scale consolidants were tested: lignin nanoparticles (LNPs) obtained form beech wood *via* a non-solvent method involving dialysis; bacterial nanocellulose (BC) obtained from cultures fed with agro-alimentary waste; cellulose nanocrystals (CNC) chemically extracted from native cellulose. Waterlogged archaeological wood samples of different species (oak, elm, stone pine, and silver fir) characterized by different levels of degradation were impregnated with the consolidants. The treatments efficiency was evaluated in terms of macroscopic observation of treated samples, anti-shrink efficiency (ASE) and equilibrium moisture content (EMC). The results obtained for the three consolidants showed substantial differences: LNPs and CNCs penetrated only about a millimeter inside the treated wood, while BC formed a compact layer on the surface of the cell walls throughout the thickness of the samples. In spite of successful BC penetration, physical evaluation of treatment efficiency showed that BC nanoparticles did not obtain a satisfying consolidation of the material. Based on the reported results more focused test protocols are optimized for future consolidation experiments.

## Introduction

The European standard EN335 (2013) (Conservation of cultural heritage—Guidelines for management of waterlogged wood on terrestrial sites of archaeological significance) defines waterlogged archaeological wood as: “wood whose structure has been filled with water through the sustained inclusion in a water saturated environment.” This wood comes from submerged archaeological sites (in lake, sea, river, or wetland) or from land waterlogged sites. Generally, waterlogged wooden artifacts preserve their original size and shape but often they undergo severe cell wall decay due to chemical and biological factors. The pH and the salinity of water as well as the chemical nature of sediments (Hedges, 1990; Unger et al., 2001; Pearson, 2014) together with the action of biological degraders (e.g., erosion and tunneling bacteria and soft rot fungi) (Blanchette et al., 1989) affect the wood causing a more or less severe mass loss and increased porosity and permeability which lead to a spongy and weakened material.

Drying waterlogged archaeological wooden artifacts is necessary for the musealization but it is always a high risk procedure because it could cause severe shrinkages and collapses. Thus, conservation practices focus not only on removing water from wood, but also on consolidating the degraded cell walls aiming for stabilized shape and size of the artifact as well as to enable it to withstand the conditions of the future preservation environment (Grattan and Clarke, 1987). An effective consolidant confers on wood a good stabilization by using the minimal amount of product while being stable to variations in relative humidity. However, these are not the only features required from an effective consolidant, conservation history highlighted no treatment exists which is able to preserve an artifact forever. Thus, all the materials used in restoration practices must be removable or, at least, leave the object retreatable. Several materials have been tested and used for the consolidation of waterlogged archaeological wood (for a review see Christensen et al., 2012). By now, the most used consolidants are polymers (e.g., polyethilene glicol, PEG), sugars (e.g., lactitol), or resins (e.g., Kauramin) (Unger et al., 2001; Christensen et al., 2012).

The use of nanomaterials in the conservation of Cultural Heritage expanded during the last few decades (Baglioni et al., 2015). Different kinds of nanoparticles were mainly used to clean and consolidate wall paintings. Tests with nanosols of silica and alkaline nanoparticles were conducted to consolidate wood artifacts and to deacidify waterlogged archaeological wood (Chelazzi et al., 2006; Mahltig et al., 2008). Even if Cipriani et al. (2010) tested cellulose derived consolidants and Christensen et al. (2012) report of consolidation attempts made with cellulose whiskers, no additional literature is available about the consolidation of waterlogged archaeological wood with nanomaterials obtained from renewable sources.

The present work is aimed to find bio-inspired consolidants that could substitute the chemicals currently in use. In particular, focusing on the concepts of bioeconomy and circular-economy, rarely applied until now to the filed of Cultural Heritage, the study tested three cellulose and lignin nano-scale consolidants: lignin nanoparticles (LNPs), bacterial nanocellulose (BC), and cellulose nanocrystals (CNCs).

LNPs can be synthesized from technical lignin obtained as a by-product of paper industry or second generation ethanol biorefineries and have been proposed for a wide range of applications as e.g., active components or fillers in polymer blends introducing lignin properties like anti-oxidant activity or UV protection on a nano-scale level (Beisl et al., 2017).

Nanocellulose can be obtained by processing plant cellulose or as a product of primary metabolic processes of certain bacterial strains (Kargarzadeh et al., 2017). Bacterial cellulose (BC), is produced by the fermentation of *Gluconoacetobacter* species, which can produce high aspect ratio (length to diameter) nanofibers, with three-dimensional porous networks, retaining the highest purity. Because of its hydrophilic nature (99% of the constituents is water), flexibility, non-toxicity, good biocompatibility, and wide availability BC has been extensively used in diverse fields from food and paper industry to biomedical applications (Kunjalukkal Padmanabhan et al., 2017).

CNCs, also known as nanocrystalline cellulose, nanowhiskers, nanorods, and rod-like cellulose crystals, are obtained by hydrolysis with highly concentrated acids (6–8 M) and high-power mechanical or ultrasonic treatments of the crystalline domains of cellulose nanofibrils. Their dimensions and the degree of crystallinity depend on the cellulose source and extraction conditions (Abdul Khalil et al., 2014; Kargarzadeh et al., 2017).

## Materials and Methods

### Nanoparticles

Three different nanoparticles were tested as consolidants of waterlogged archaeological wood: lignin nanoparticles (LNPs), bacterial nanocellulose (BC), cellulose nanocrystals (CNCs).

LNPs were obtained from beech wood (*Fagus sylvatica* L.) from the Cimini Mountains in Lazio region, Italy (Piangoli Legno SNC, Soriano nel Cimino, VT). They were produced applying the “anti-solvent procedure” on acidolysis lignin using DMSO as lignin solvent, as reported in Zikeli et al. (2018). The obtained LNPs are hollow, sphere-shaped particles with diameters ranging between 40 and 120 nm.

BC was provided by Biofaber srl (Italy). It was obtained by fermentation of agro-alimentary waste, in accordance with Pal et al. (2017), in the form of hydrogel pellicles that were washed with distilled water and boiled in 0.5 M sodium hydroxide solution in order to purify the samples. Pellicles were dried at 60°C to get dried BC membranes and then were grinded with a rotary mill in order to obtain cellulose nanospheres.

The BC prepared for the test had never been studied before, so morphological characterization of BC spay-dried on a stub was performed on a Zeiss (Sigma VP; Carl Zeiss, Germany) FESEM.

CNCs were supplied by CelluForce (Montreal, Canada). CelluForce NCC® is a cellulose hydrogen sulfate sodium salt, provided as a spray dried powder, white in color. The CNCs are spindle shaped crystals, 2.3–4.5 nm in diameter and 44–108 nm in length (from CelluForce product specifications).

### Wood Samples

Wood samples were obtained from two archaeological sites: Isola Sacra (Fiumicino, Italy) and the ancient port of Neapolis (Naples, Italy). The wood coming from Fiumicino belongs to the shipwreck named Isola Sacra 1 (sample P2550), an horeia-type vessel dated before the first half of the 3rd century AD and excavated in 2011 (Boetto et al., 2017). The wood from Naples pertains the shipwreck F (samples FTdis 4, Ftdis 9, and FTdis 48) (Di Donato et al., 2018), and a wooden pier (2514.28, 2514.30, and 2514.34) both dated to the roman age. The remains were recovered from the excavation area of Piazza Municipio in 2015 and 2018, respectively. After the recovery, the wood was stored in the biology laboratory of the Istituto Superiore per la Conservazione e il Restauro (IsCR). During all the storage period the fragments were left completely soaked to maintain the state of maximum water content.

From the wood samples 47 blocks were obtained with dimensions 2.5 × 2.5 × 1.5 cm and 3.0 × 1.5 × 1.5 cm, depending on the availability of the material. Ten blocks were used for each consolidant to be tested, the remaining were used as freeze- (7) and air-dried (10) not-consolidated controls.

Each sample was marked with an acronym indicating the consolidant (L, lignin nanoparticles; BC, bacterial cellulose; CNC, cellulose nanocrystals) or the drying method for controls (F, freeze-drying; A, air-drying), and the wood type (S, softwood; H, hardwood) followed by sequential numbers (Table 1).

**Table 1:** The utilized samples, type of wood, archaeological sample and consolidant, or drying method.

**Sample name**	**Wood**	**Archaeological sample**	**Consolidant/ drying method**
LS1	Softwood	P2550	Lignin nanoparticles
LS2		FTdis 4	
LS3		FTdis 48	
LH1–LH2–LH3	Hardwood	FTdis 9	
LH4–LH5		2514.28	
LH6		2514.30	
LH7		2514.34	
BCS1	Softwood	P2550	Bacterial cellulose
BCS2		FTdis 4	
BCS3		FTdis 48	
BCH1–BCH2–BCH3	Hardwood	FTdis 9	
BCH4–BCH5		2514.28	
BCH6		2514.30	
BCH7		2514.34	
CNCS1	Softwood	P2550	Cellulose nanocrystals
CNCS2		FTdis 4	
CNCS3		FTdis 48	
CNCH1–CNCH2–CNCH3	Hardwood	FTdis 9	
CNCH4–CNCH5		2514.28	
CNCH6		2514.30	
CNCH7		2514.34	
FS1	Softwood	P2550	Freeze-drying
FS2		FTdis 4	
FS3		FTdis 48	
FH1	Hardwood	FTdis 9	
FH2		2514.28	
FH3		2514.30	
FH4		2514.34	
AS1–AS2	Softwood	P2550	Air-drying
AS3		FTdis 4	
AS4		FTdis 48	
AH1–AH2	Hardwood	2514.28	
AH3–AH4		2514.30	
AH5–AH6		2514.34	

### Wood Characterization

Before the beginning of consolidation procedures the wood species were identified and the state of preservation of the material was characterized by micro-morphological and physical analyses according to the Italian standard and to the most used protocols (Jensen and Gregory, 2006; Schwarze, 2007; UNI11205, 2007; Capretti et al., 2008; Romagnoli et al., 2018).

The identification of wood species and micro-morphological examinations were carried out on thin sections (10–20 μm) cut in the three anatomical planes (cross, longitudinal-radial and longitudinal-tangential) by mean of transmission light microscopy (DMRB, Leitz) as prescribed by UNI11118 (2004). Samples were cut by hand with a razor blade or using a cryo-microtome (Cryostat CM 1900, Leica). To identify the wood species the observed anatomical features were compared to literature works (Jacquiot, 1955; Jacquiot et al., 1973; Schweingruber and Baas, 1990).

To highlight the presence of micro-organisms the sections were stained with an aqueous solution of 1% w/v methylene blue in 50% lactic acid. Microbial decay was evaluated using both bright-field and polarized light microscopy in order to identify decay patterns and highlight the loss of crystalline cellulose.

To assess the decay by means of physical tests wood blocks were weighted and their volume was defined by the water displacement method. Weight was measured again after the samples were dried in an oven at 103 ± 2°C up to a constant weight. The physical parameters used for the characterization are reported in Table 2. Reference values for basic density of non-decayed wood were taken from the literature (Giordano, 1986; Capretti et al., 2008; Macchioni et al., 2012).

**Table 2:** Physical parameters used to asses the wood decay.

**Physical parameter**	**Unit**	**Formula**
Basic density (D_bd_)	g × cm^−3^	$D_{bd}=\frac{M_{0}}{V_{f}}$
Maximum water content (MWC)	%	$MWC=\frac{M_{f}-\;M_{O}}{M_{O}}$
Residual density (RD_b_)	%	$RD_{b}=\frac{D_{bd}}{D_{b}}$

*M_0_, anhydrous mass; M_f_, mass at maximum water content; V_f_, volume at maximum water content; D_b_, basic density of non-decayed wood*.

### Methods Development of Impregnation and Wood Drying

Impregnation baths containing the nano-scale consolidants were prepared. LNPs' bath was obtained adding the nanoparticles to deionised water up to a concentration of 5 mg/ml. BC suspended in deionised water tended to precipitate, so to obtain a stable suspension several attempts were made. Following the suggestions of the Biofaber team, Tween 20, sucrose, polyethylene glycol 400 (PEG400), oil or a mix of them were added to the BC (concentration 5%), the suspensions were homogenized with the help of a magnetic shaker or a sonicator. The details of the performed tests are reported in Table S1. At the end of every test a more or less thick layer of BC had precipitated at the bottom of the container, except for the suspension obtained with procedure reported as T2 in Table S1 (0.5% Tween 20 + 1% Cinnamon oil + deionised water, sonication 50% amplitude−1 h, 5% BC, magnetic shaking 500/600 rpm−1 h), which in consequence was selected as the procedure to prepare the impregnation baths.

To obtain a liquid suspension of CNCs, 3% of powdered crystals were added to deionised water and shaken on a magnetic shaker for 1 h. By adding a greater amount of CNCs the suspension became a gel and thus not suitable for the consolidation treatment.

To perform the consolidation tests, the waterlogged archaeological wood blocks were completely soaked in the baths for 1 month at room temperature. After the treatment, the blocks were frozen (−20°C) and then freeze-dried following the procedure settled in the IsCR restoration laboratory *Excavated Organic Materials*. The lyophilizer (LIO 2000PNS) settings were: chamber temperature −30°C; condenser temperature −50°C; chamber vacuum 2 Pa. The wood temperature was monitored through the use of thermocouples positioned on the samples' surface. The pressure was raised up to room pressure and the blocks were removed from the lyophilizer when reaching a temperature of 18–20°C.

F control blocks were kept completely soaked in deionised water and were successively frozen and freeze-dried under the same condition as treated samples. A controls samples were air-dried at room temperature.

During the consolidation treatments different problems arose for the three tested consolidants. Soon after the beginning of consolidation procedure LNPs and BC precipitated on the bottom of the container. Therefore, to keep the nanoparticles suspended the impregnation baths were gently shaken on a magnetic stirrer for the rest of the test. Additionally, the wood blocks were periodically rotated in order to avoid nanoparticles deposition on their upper surface. In spite of all the precautions taken, at the end of the treatments a more or less thick layer of LNPs and BC was present on the wood (Figures S1A,B). Those nanoparticles were removed with a soft brush before freezing the blocks.

After 1 week of consolidation test, the CNCs impregnation bath turned into a gel (Figure S1C). A test carried out on a small aliquot of the gel showed that to regain a liquid bath the addition of the double volume water was necessary. This solution was not considered in order to avoid an excessive dilution of the consolidant. At the end of the treatment, the wood blocks were extracted from the gel and the residual CNCs on the wood surface were removed with a soft brush.

### Evaluation of the Efficacy of the Treatments

A first evaluation of the efficacy of the treatments was obtained by macroscopic observation of the samples. Color, shape, presence of openings, and consistency were observed.

The penetration of the nanoparticles inside the wood tissues was evaluated by examining gold sputter-coated sections of treated blocks (LH5, BCH5, and CNCH5) and freeze-dried control (FH2) with a Scanning Electron Microscope (Zeiss EVO 60) in secondary electrons mode. For each sample three sections were observed: external cross section, internal cross section, and internal radial section. The internal sections were obtained splitting the block by hand, without the use of blades.

#### Anti-Shrink Efficiency

In order to evaluate the dimensional variations of treated wood, all the six sides of treated blocks, freeze- and air-dried controls were scanned on high definition scanner (HP scanjet G3010), together with a measuring ruler, before the consolidation treatment, and after the drying procedure (wood equilibrated at 20°C, 50% RH). Using an image processing software (ImageJ 1.52n) the cross section surface and the average height of each block were measured. The obtained values were used to calculate the blocks' volume (V_W_, volume of waterlogged blocks; V_D_, volume of treated blocks and controls after drying).

For each block, the percentage shrinkage, S, was calculated following the equation:

$$\begin{array}{l} S\left(\%\right)=\frac{V_{W\;}-\;V_{D}}{V_{W}}*100 \end{array}$$

The S values obtained for each treated sample were used to determine the anti-shrink efficiency (ASE) with respect to freeze-dried (ASE_F_) and air-dried (ASE_A_) controls following the equations:

$$\begin{array}{l} ASE_{F}\left(\%\right)=\frac{S_{F}-S_{T}}{S_{F}}*100\text{ and}\\ \;ASE_{A}\left(\%\right)=\frac{S_{A}-S_{T}}{S_{A}}*100 \end{array}$$

Where S_F_ and S_A_ are the shrinkages of freeze- and air-dried controls, respectively, and S_T_ is the shrinkage of treated blocks.

The ASE_C_, expressed as the percentage of shrinkage that has been suppressed by freeze-drying treatment as compared to the shrinkage of air-dried controls, was calculated as follows:

$$\begin{array}{l} ASE_{C}\left(\%\right)=\frac{S_{A}-\;S_{F}}{S_{A}}*100 \end{array}$$

#### Equilibrium Moisture Content

To evaluate the effect of the different consolidation treatments with respect to moisture absorption the equilibrium moisture content (EMC) was determined. The test was performed on the treated wood blocks and freeze-dried controls not used for SEM observations. The obtained results were compared to sound wood EMC from literature (Giordano, 1981) and to values obtained for sound wood controls. The latter, four blocks of silver fir and six blocks of holm oak, 2.0 × 2.0 × 0.5 cm in dimensions, were marked with a progressive number.

The samples were put in a climatic chamber at 20°C and the RH was varied according to the sequence 10, 35, 45, 55, 65, 85% reaching in each step the EMC (Giordano, 1981). The EMC for the RH value of 100% was obtained by putting the wood blocks in a closed container in presence of liquid water. At the end of the RH cycle, the samples were completely dried in a oven at 103 ± 2°C up to a constant weight.

## Results and Discussion

### Bacterial Cellulose Nanoparticles

**Figure 1:**
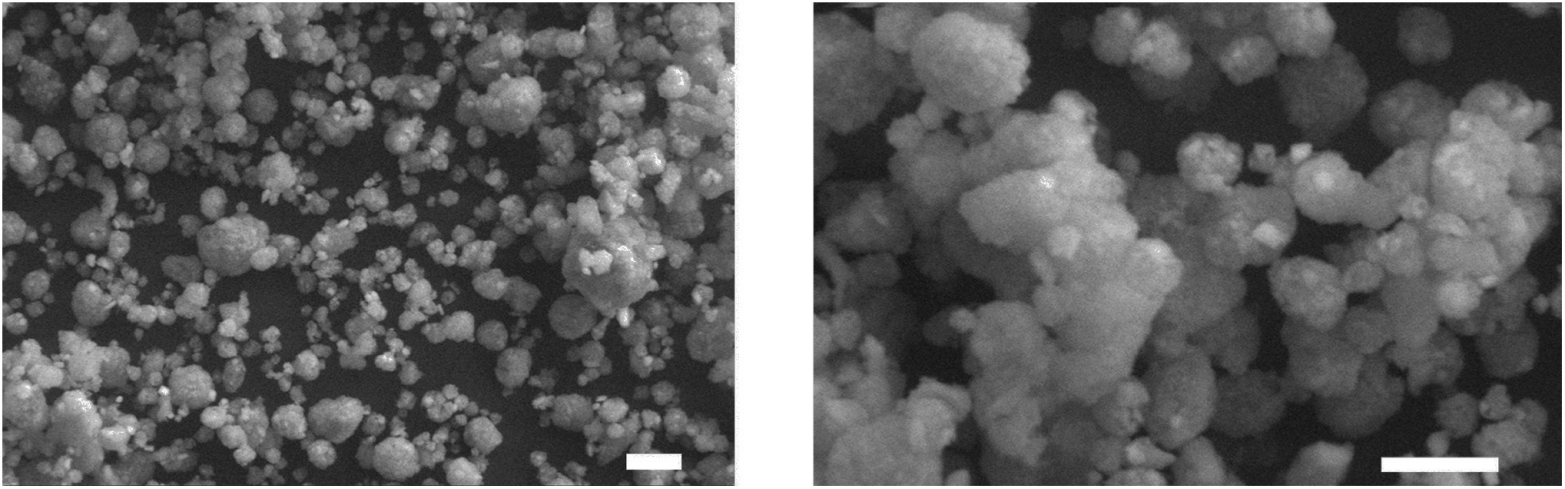
SEM images of the spray dried bacterial cellulose. Scale bars 10 μm.

**Figure 2:**
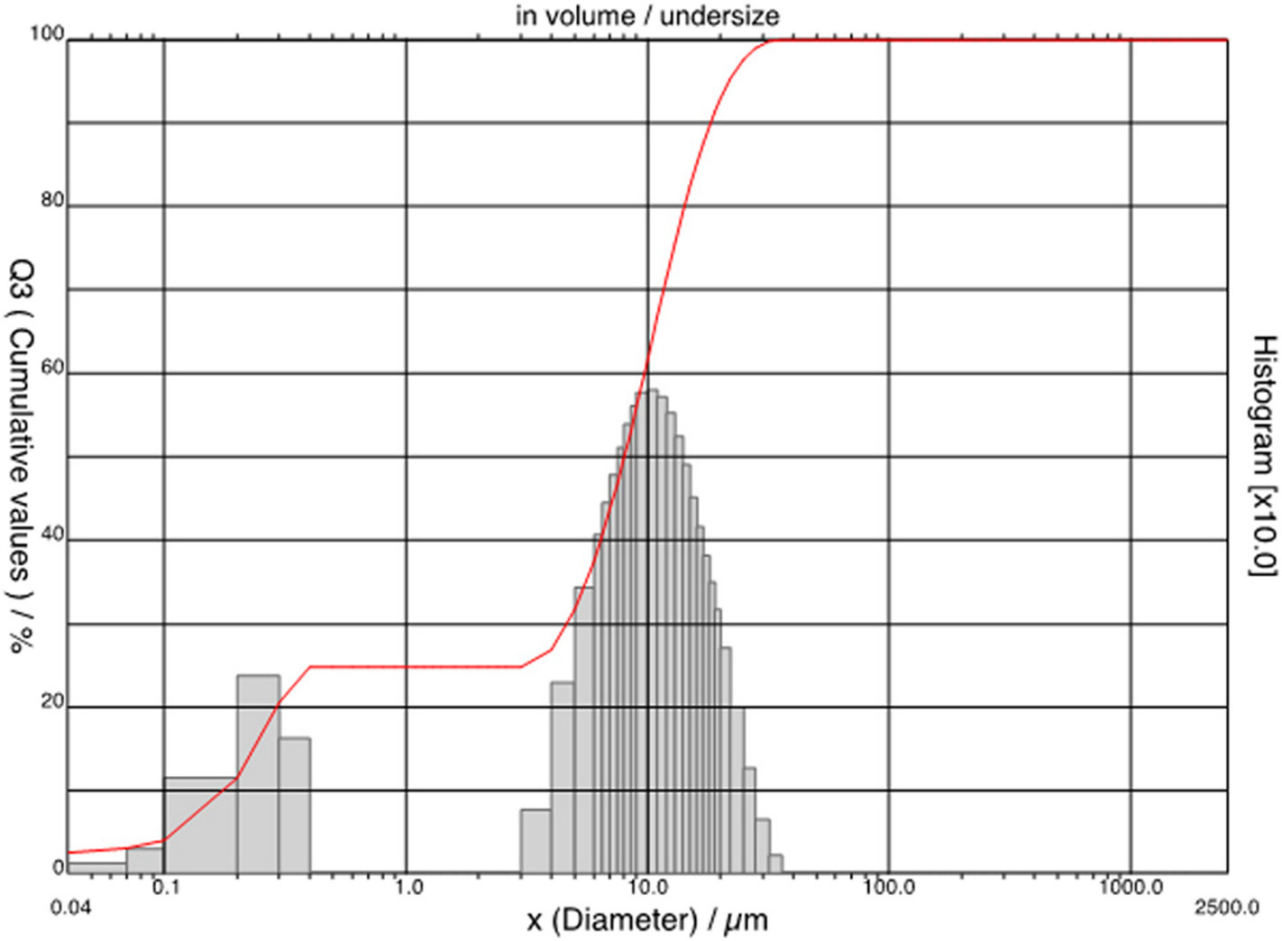
Size distribution of the spray dried bacterial cellulose.

SEM observations of bacterial nanocellulose allowed for characterizing the consolidant. Most part of the observed nanoparticles was more or less spherical in shape (Figure 1), in some cases small, irregularly shaped particles were observed. The size distribution (Figure 2) showed that the nanoparticles could be divided into two dimensional classes with diameter ranges of 0.04–0.50 μm and 3–50 μm. Most part of the BC particles had a diameter of ca 10 μm.

### Wood Characterization

Results regarding wood species identification and physical analyses are reported in Table 3. The softwood samples were referred to stone pine (*Pinus pinea* L.), silver fir (*Abies alba* Mill.) and cypress (*Cupressus sempervirens* L.). Regarding the hardwood samples, the sample FTdis 9 was identified as elm (*Ulmus* sp.) while the blocks obtained from the wooden pier belonged to the evergreen oak group and can be probably attributed to holm oak (*Quercus ilex* L).

**Table 3:** Results of physical characterization of wood samples.

**Archaeological sample**	**Wooden species**	**D_**bd**_ (g × cm^**−3**^)**	**MWC (%)**	**RD_**b**_ (%)**
P2550	*Pinus pinea*	0.31	254	61
FTdis 4	*Abies alba*	0.41	193	108
FTdis 9	*Ulmus* sp.	0.34	250	61
FTdis 48	*Cupressus* sp.	0.25	331	41
2514.28	*Quercus* cfr. *ilex*	0.15	616	20
2514.30	*Quercus* cfr. *ilex*	0.12	741	17
2514.34	*Quercus* cfr. *ilex*	0.14	648	19

Micro-morphological analyses of wood thin sections allowed for observing the microbial decay of the tissue which was mainly ascribed to erosion bacteria (Figure S2A), even though fungal hyphae and spores were observed in several sections (Figure S2B). The observations carried out in polarized light showed a severe loss of the birefringence typical of cellulose (Figures S2C,D), a sign that the crystalline structure of the polymer was almost completely lost due to microbial attack.

The physical parameters used to assess the decay showed different levels of degradation for the selected woods. The highest MWC values were recorded for the holm oak samples (616, 741, and 648% respectively). For the three poles the residual density ranged between 17 and 20%. According to the degradation classes established by De Jong (Grattan and Clarke, 1987) and by McConnachie et al. (2008) these samples can be considered as highly degraded.

The stone pine samples were the best preserved of the analyzed woods with MWC value of 254% and RD_b_ of 61%. Cypress showed an intermediate level of degradation with an MWC of 331% and an RD_b_ of 41%. According to De Jong these woods belong to the intermediate class of degradation.

Silver fir and elm samples deserve a special mention. Based on the MWC values (193 and 250%) these samples resulted as the best preserved of all analyzed wood. However, the fir RD_b_ value (108%) and the fact that elm was much more degraded (spongy tissue, not resisting to the cut) with respect to wood with similar MWC indicated that the results could not be considered as reliable. The wood was characterized by the presence of several shipworm tunnels filled with sediments. During the cleaning operations carried out before the tests it was not possible to remove all the sediments from the samples, especially from the innermost part. It is known that the presence of sediments and/or mineral depositions inside archaeological waterlogged wood influences the RD_b_ values (Schniewind, 1990), so the unreliable results obtained for the physical analyses must be attributed to this cause.

### Evaluation of Efficacy of Consolidation Treatments

Macroscopic observation of treated samples and comparison between consolidated and un-consolidated wood allowed for a first evaluation of the effect of the respective consolidation treatments.

**Figure 3:**
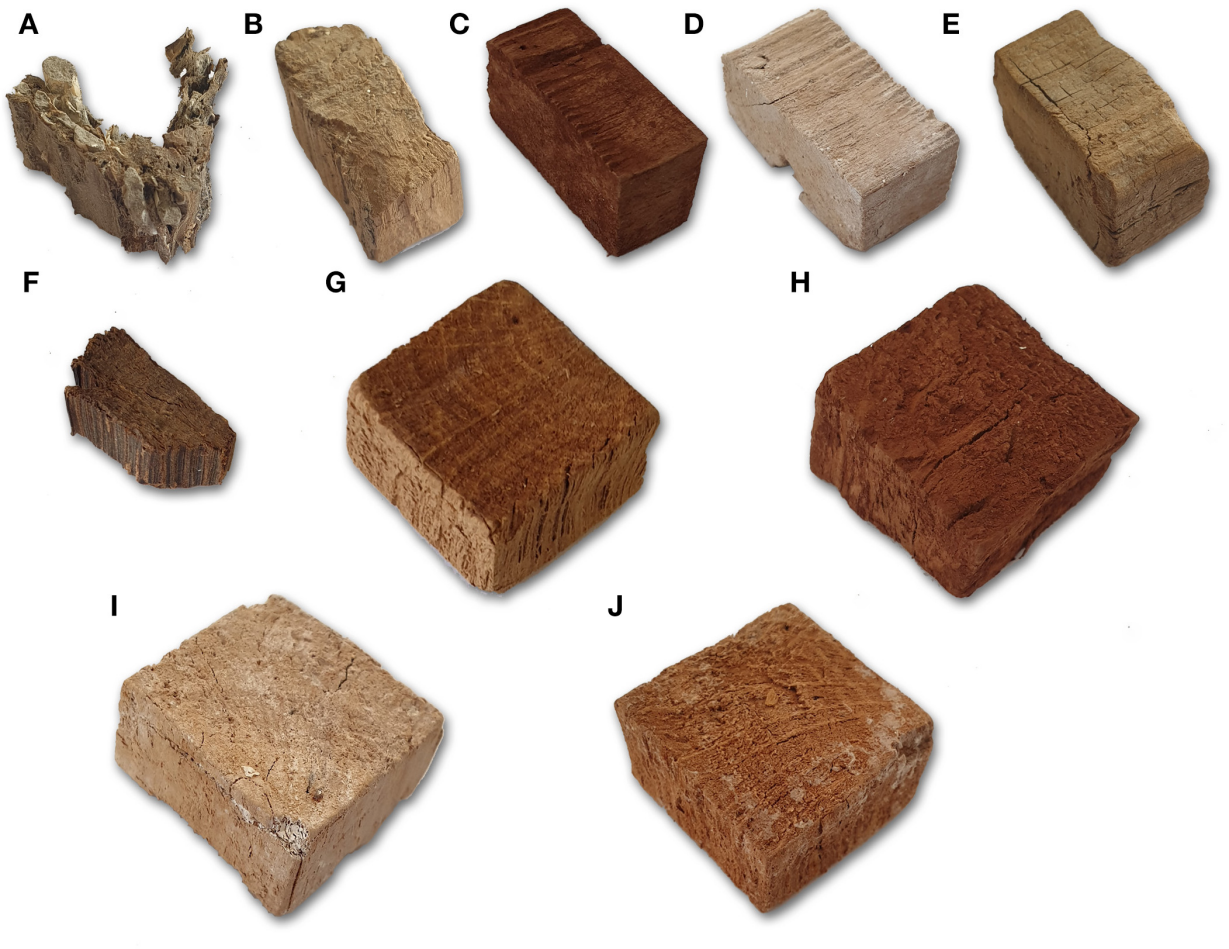
Aspect of selected treated blocks and controls. **(A)** AS3—silver fir; **(B)** FS3—cypress; **(C)** LS1—stone pine; **(D)** BCS1—stone pine; **(E)** CNCS3—cypress; **(F)** AH3—elm; **(G)** FH4—holm oak; **(H)** LH4—holm oak; **(I)** BCH6—holm oak; **(J)** CNCH7—holm oak.

In contrast to air-dried controls, all treated samples and freeze-dried controls apparently maintained the original shape and no dimensional differences were perceivable by naked eye between differently consolidated blocks and freeze-dried controls (Figure 3 and Figures S3, S4). In few cases, of both consolidated and freeze-dried blocks, small pieces of wood had detached during consolidation or drying procedure and small openings were present on the samples' surfaces.

Wood color and aspect were widely affected by consolidation treatments. LNPs treated samples resulted in dark brown color and a fine brown powder was present on the sample surface, even after freeze-drying. BC consolidated blocks were whitish or yellowish in color. When broken for SEM observation, it was evident that the color change had occurred throughout the block thickness. Finally, CNCs treated samples showed a color similar to the controls, but a thin, colorless film was present on almost all the surface of the blocks. That film was easily removable by touching the wood.

**Figure 4:**
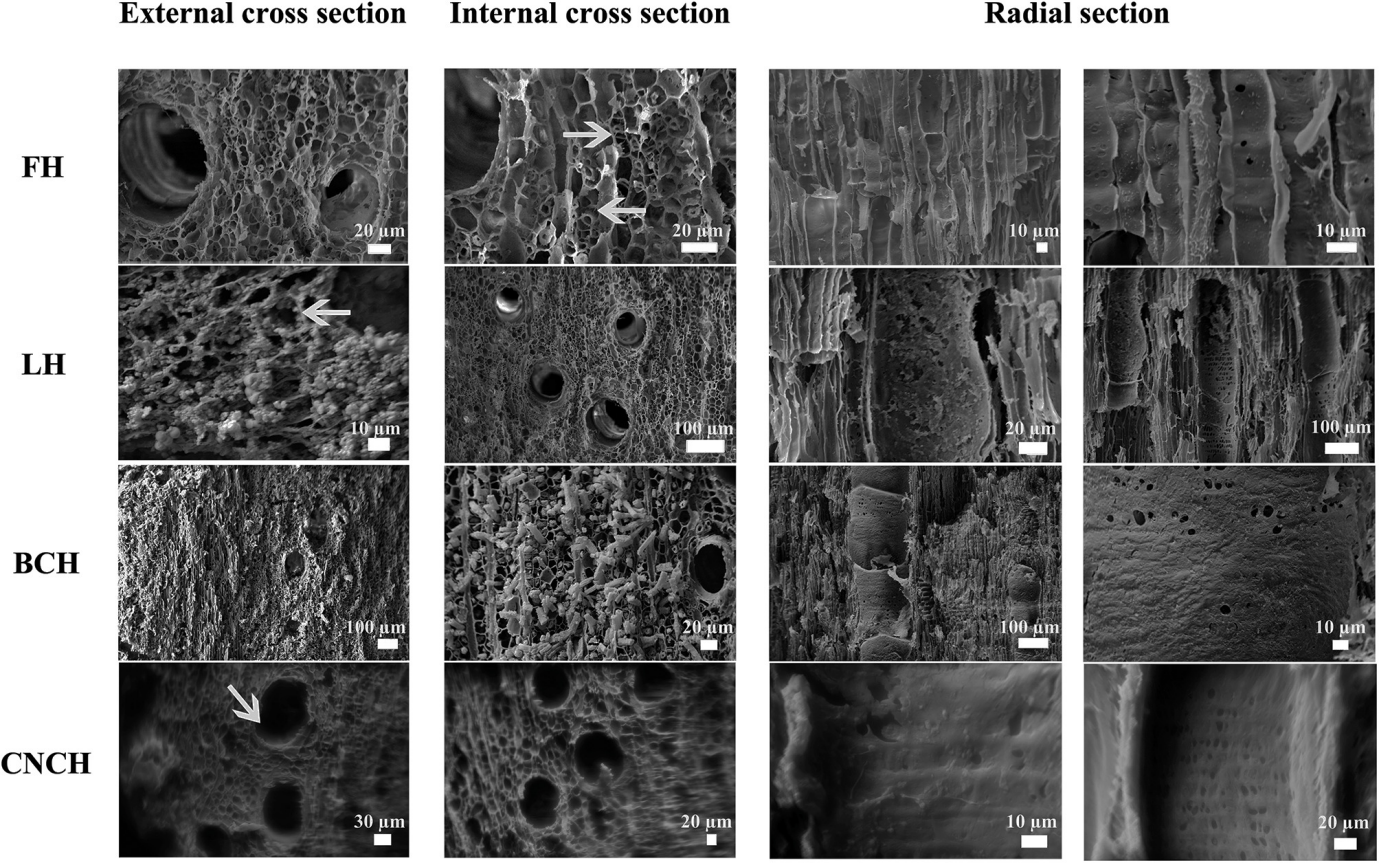
SEM images of freeze-dried control (FH2) and treated blocks (LH5, BCH5, and CNCH5). **FH**—external and internal cross sections: cell lumens are empty, secondary cell walls appear almost completely detached or linked by thin filaments (arrows) to the middle lamella; radial section: vessel and fiber walls appear smooth and the pits are well visible. **LH**—external cross section: a layer of nanoparticles is present on the surface, sometimes a coat of nanoparticles is visible inside the cells, all around the lumen (arrow); internal cross section: the cell lumens are empty, no nanoparticles are visible; radial section: LNPs are present inside some of the vessels forming a layer on the cell walls for the first 400 μm, the other vessels and the wood fibers are empty. **BCH**—external cross section: sporadic deposits of nanoparticles are visible on the surface, some of the cell lumens are filled with consolidant (well visible inside vessels); internal cross section: the degraded secondary cell walls appear as “tubes” coming out of the section; radial section: a homogeneous and compact layer of BC is visible on the cell walls of both vessels and fibers, covering most part of the pits. **CNCH**—external cross section: the cell lumens are empty and only in some cases a thin layer of CNCs is visible on the surface of cell walls (arrow); internal cross section: the cell lumens are empty; radial section: nanoparticles formed a compact and homogeneous coat almost completely covering the pits, at depths >1.3 mm the CNC layer is no more visible, the cell walls appear smooth and the pits are well visible.

SEM observations of the treated wood samples allowed for observing how the nanoparticles had penetrated inside the wood tissues. Figure 4 reports the images of external and internal cross sections and radial sections of all the observed blocks. The analysis of a control un-consolidated block showed that in the cross sections, both external and internal, cell lumens were empty and in several cases the secondary cell walls appeared mostly detached from the middle lamella (ML) with only thin filaments connecting the cell wall to the ML. In radial section the vessel and fiber walls appeared smooth and pits were well visible.

In the LNPs treated block a layer of nanoparticles was observed on the external cross section. Most of the cell lumens were covered with compact deposits of LNPs. In other cases, a coating of nanoparticles was well visible covering the whole cell lumen wall. In the internal cross section the cell lumens appeared empty and no nanoparticles were visible. Observing the radial section, it resulted that LNPs penetrated inside some vessels up to a depth of ca 1.2 mm and formed a more or less compact and irregular layer covering the cell walls for the first 400 μm. Only few sporadic particles penetrated deeper into the wood tissue and a part of wood vessels and fibers appeared completely empty.

On the external cross section of the BC treated block, only few sporadic deposits of nanoparticles were observed. Most of the cell lumens were filled with consolidant, the rest seemed empty and the secondary cell walls detached from the ML were well visible. Observing the internal cross section of the block, in most cases the degraded secondary cell walls appeared as “tubes” coming out of the section. In other cases, only the ML was present and the cell lumen was empty. In the radial section, a homogeneous and compact layer of BC was observed on the cell walls of both vessels and fibers throughout the thickness of the block, covering most part of the pits.

In the external cross section of the CNCs treated block, the cell lumens were empty and only in some cases a thin layer of CNCs was observed on the surface of the cell walls. Nanoparticles had penetrated up to a depth of ca 1.3 mm forming a compact and homogeneous coating that covered almost completely the pits, as visible in radial section. At greater depths, the CNCs layer was not visible anymore thus cell walls appeared smooth like observed for the control sample and all pits were well visible. The empty cell lumens and the absence of nanoparticles coating in the internal cross section confirmed the scarce consolidant penetration.

As shown, the results obtained with the three nano-scale consolidants were quite different. Shape and dimensions of the nanoparticles did not affect the treatment. In fact, CNCs showed almost the same penetration behavior as LNPs despite being much smaller and having a needle-like shape that initially suggested a greater ease of penetration inside the cells. In contrast, the BC nanoparticles penetrated evenly inside the material despite a more irregular shape and larger particle size.

The scarce LNPs penetration may be mainly linked to the nanoparticles hydrophobicity that lead to their aggregation and precipitation during the consolidation procedure and could present an obstacle for the penetration into the water-soaked samples. Further, substances present in the sediments inside the wood could have influenced the suspension's pH accelerating this phenomenon.

As mentioned above, the CNCs impregnation bath turned into a gel during the treatment, indicating that nanoparticles interacted with each other forming the gel matrix and thus preventing successful penetration inside the sample material. BC nanoparticles showed no interaction with each other and penetrated inside the wood not filling up the voids of the structure but creating a compact layer on cell walls. This observation suggested that the consolidant interacted with OH groups available from cellulose and/or degraded lignin in the cell walls. The presence of “tubes” coming out the internal cross section, not observed in the control block, suggested that in the block treated by BC the nanoparticles had impregnated the detached cell walls and thus prevented breaking of the cell walls during splitting of the block. Nevertheless, the consolidation was not successful enough to allow the secondary walls to re-join the ML. Indeed, while in the control block in some cases subtle connections were observed between ML and secondary wall, in the BC treated wood these connections got lost. Probably, the BC present in the walls suffered from stress during freeze-drying that broke these links. The absence of this desired consolidant effect explains the fragile appearance of the treated wood, material loss and creation of openings in the blocks during treatment.

The nanocellulose shape affects how it interacts with the consolidated material. BC and CNCs penetrate inside the wood and precipitate on the cell walls' surface creating hydrogen bonds with the wall components. Christensen et al. (2012) report of an attempt of consolidation of waterlogged archaeological wood by using cellulose whiskers, rod-like nanoparticles 15 nm in diameter and 200–300 nm in length. The authors observed also in their case problems with flocculation and an altered wood aspect caused by the superficial adhesion of nanoparticles. However, a consolidation effect was observed, the whiskers acted as gap fillers interacting between each other and creating an open net-structure inside wood tissues.

#### Anti-Shrink Efficiency

The values of ASE_F_, ASE_A_, and ASE_C_ are shown in Table 4. The percentage of shrinkage suppressed by the consolidation and freeze-drying process with respect to air-dried controls, ASE_A_, was higher than 50% for all samples with the only exception of block CNCS1. More specifically, for all the consolidation treatments ASE_A_ obtained for hardwood ranged between 80 and 88%, while the values obtained for softwood showed a greater variability between the different consolidants as well as among the blocks treated with the same nanoparticles. In fact, for LNPs treated blocks the registered values ranged between 51 and 69%, for BC treated blocks between 55 and 94% and for CNCs treated samples between 37 and 66%.

**Table 4:** Anti-shrink efficiency of the consolidation treatments with respect to freeze-dried (ASE_F_) and air-dried (ASE_A_) controls and anti-shrink efficiency of freeze-drying procedure with respect to air-drying (ASE_C_).

**Sample**	**V_**W**_ (mm^**3**^)**	**V_**D**_ (mm^**3**^)**	**S (%)**	**ASE_**F**_ (%)**	**ASE_**A**_ (%)**	**ASE_**C**_ (%)**
LS1	5861.81	5449.78	7.02	17	60	–
LS2	5403.67	4196.31	22.34	−96	51	–
LS3	4121.53	3567.99	13.43	2	69	–
LH1	5608.21	5001.67	10.81	40	–^*^	–
LH2	6646.23	5704.47	14.17	21	–	–
LH3	5702.48	4981.53	12.64	30	–	–
LH4	11070.66	9921.53	10.38	43	88	–
LH5	9264.25	7679.67	17.10	7	80	–
LH6	9917.58	8714.65	12.13	28	86	–
LH7	10308.65	9062.89	12.08	36	86	–
BCS1	6320.24	5918.37	6.36	25	55	–
BCS2	5317.96	5164.92	2.88	75	94	–
BCS3	3314.78	2851.31	13.98	−2	68	–
BCH1	6625.52	5748.63	13.23	26	–	–
BCH2	6390.79	5620.76	12.05	33	–	–
BCH3	6342.45	5479.22	13.61	24	–	–
BCH4	9015.00	7552.07	16.23	12	81	–
BCH5	10739.92	9173.65	14.58	21	83	–
BCH6	9509.61	8041.09	15.44	9	82	–
BCH7	9736.55	8260.79	15.16	20	82	–
CNCS1	7580.56	6754.18	10.90	−28	37	–
CNCS2	6322.14	5337.42	15.58	−37	66	–
CNCS3	5700.13	4781.69	16.11	−18	63	–
CNCH1	5377.48	4854.08	9.73	46	–	–
CNCH2	5165.71	4516.12	12.57	30	–	–
CNCH3	6111.08	4970.19	18.67	−4	–	–
CNCH4	9557.40	8067.11	15.59	15	82	–
CNCH5	11624.52	10036.73	13.66	26	84	–
CNCH6	8864.71	7521.15	15.16	10	82	–
CNCH7	12059.45	10306.49	14.54	23	83	–
FS1	4482.83	4101.29	8.51	–	–	51
FS2	4402.86	3902.14	11.37	–	–	75
FS3	3178.20	2742.74	13.70	–	–	69
FH2	11569.56	9446.78	18.35	–	–	78
FH3	5413.94	4500.20	16.88	–	–	80
FH4	8646.58	7014.09	18.88	–	–	78

* *These ASE values are missing because the archaeological material was not enough to obtain an air-dried control*.

ASE_F_ compares the volumetric shrinkages of consolidated/freeze-dried blocks to those of un-consolidated/freeze-dried controls representing the contribution of the consolidants to the shrinkage suppression. The results obtained for this parameter were generally lower than those calculated for ASE_A_, and for six of the treated blocks negative values were obtained. The highest value was registered for the block BCS2 (75%), the other positive results ranged between a minimum of 2% and a maximum of 46%. The low and negative values obtained for LNPs and CNCs were obviously linked to the fact that the consolidants had penetrated only into a thin layer inside the sample material and thus did not counteract sample shrinkage. The negative value of BCS3 and the low positive values obtained for the others BC treated samples may be linked to the fact that the bacterial cellulose was not able to recreate the bonds between middle lamella and cell wall upon successful sample penetration, as mentioned above.

Regarding the high ASE_A_ values calculated comparing treated blocks and air-dried controls the effect of freeze-drying must be considered. The values of ASE_C_, representing the percentage of shrinkage suppressed by freeze-drying treatment compared to the shrinkage of air-dried controls, confirm the predominant anti-shrink efficiency of freeze-drying. In fact, for hardwood samples ASE_C_ values ranged between 78 and 80% and for softwood samples between 51 and 75%. It is known from literature (e.g., Pearson, 2014) that freeze-drying of waterlogged archaeological wood allows for avoiding collapses and reduces shrinkage conserving object shape and dimension. However, the use of lyophilization without a previous consolidation procedure leads to stress-caused cracking in the fragile material. Aspect and consistency of the nanoparticles treated blocks reflected the just mentioned problems, confirming that an effective consolidation was not achieved.

**Figure 5:**
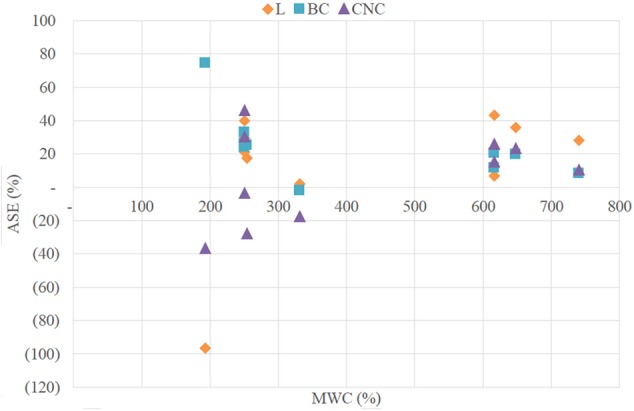
Graph of Anti-shrink efficiency (ASE) vs. MWC.

As shown in Figure 5 no relations were found between MWC and ASE.

#### Equilibrium Moisture Content

**Figure 6:**
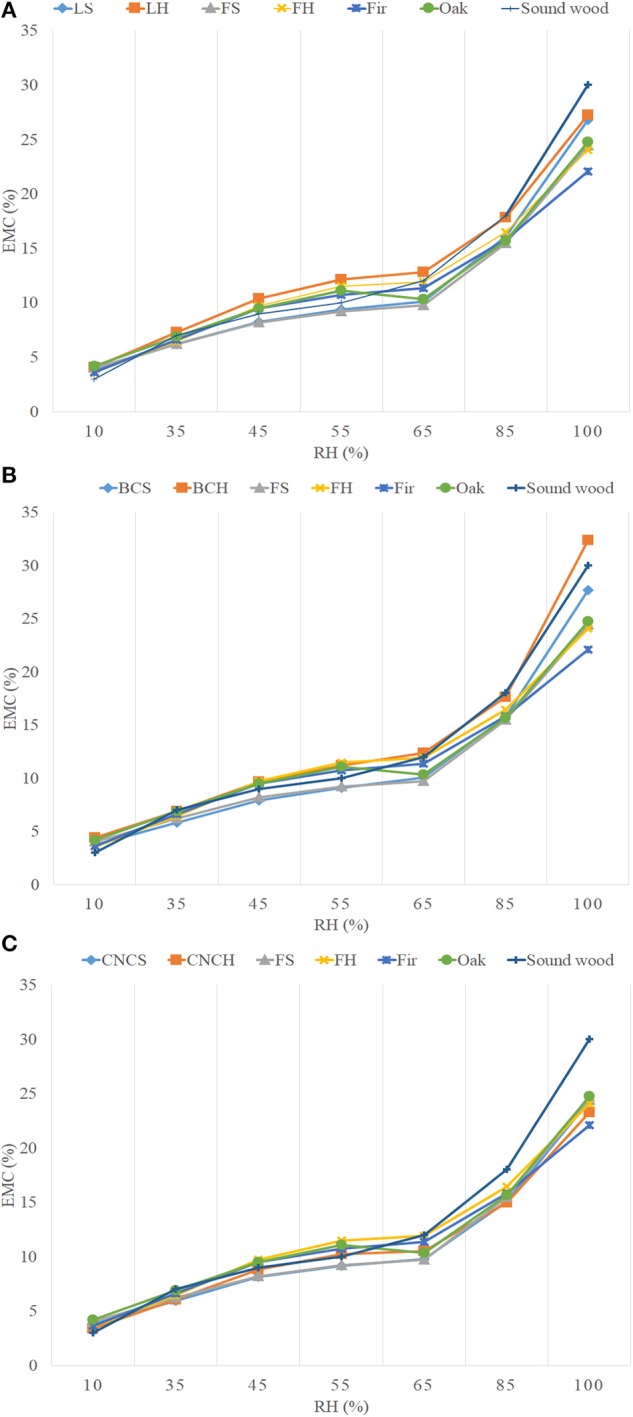
Equilibrium moisture content (EMC) trends of treated blocks compared to freeze-dried controls, sound wood controls and sound wood values obtained from the literature. **(A)** LNP; **(B)** BC; **(C)** CNC.

Table 5 and Figure 6 show the values and trends of the EMC of treated blocks compared to freeze-dried controls, sound wood controls (oak and fir) and sound wood values reported in literature. The trends, obtained by averaging the values in Table 5, showed that the EMC of sound wood controls used during the experimentation were lower with respect to literature values. This can be linked to the natural variability of wood. The values obtained for freeze-dried wood were comparable to those of sound controls for all tested RH.

**Table 5:** Equilibrium moisture content (EMC) of treated samples, freeze-dried controls, sound wood controls, and sound wood from the literature at every RH stage.

**Sample**	**EMC**						
	**10%**	**35%**	**45%**	**55%**	**65%**	**85%**	**100%**
LS1	4.43	8.05	11.24	13.24	13.35	19.67	31.83
LS2	2.84	4.08	4.55	4.85	5.38	8.69	15.87
LS3	4.52	6.59	8.92	9.97	11.55	19.90	32.72
LH1	4.71	7.76	10.84	12.45	13.03	18.04	26.65
LH2	3.29	7.03	9.53	11.13	14.24	17.61	23.74
LH3	5.26	8.44	12.12	14.17	14.53	19.91	32.49
LH4	3.55	6.81	9.95	11.74	11.76	17.30	27.25
LH5	4.29	7.20	10.46	12.14	12.29	17.72	27.57
LH7	3.18	6.43	9.61	11.18	11.07	16.52	25.92
BCS1	4.30	7.53	10.56	12.31	13.06	18.84	31.83
BCS2	2.79	3.77	4.75	5.25	5.74	9.90	17.56
BCS3	4.21	6.14	8.44	9.68	11.35	18.53	33.60
BCH1	4.72	7.48	10.38	11.97	13.03	18.03	27.55
BCH2	4.66	6.91	9.66	11.25	12.15	16.67	28.66
BCH3	4.40	6.84	9.77	11.41	12.52	17.03	25.62
BCH4	4.18	6.39	9.11	10.51	11.53	17.42	40.32
BCH6	4.16	7.01	9.64	11.16	12.79	18.62	36.22
BCH7	4.27	6.75	9.55	10.93	12.26	17.98	35.81
CNCS1	4.36	8.14	11.56	13.46	13.30	19.40	27.04
CNCS2	2.83	3.50	4.26	4.28	4.72	7.10	13.24
CNCS3	4.13	6.25	8.59	9.68	11.26	18.74	32.85
CNCH1	2.69	4.61	6.49	7.60	8.34	10.93	15.99
CNCH2	2.95	4.53	6.40	7.42	8.65	11.28	15.34
CNCH3	4.83	8.08	11.27	12.91	13.15	18.61	28.81
CNCH4	3.35	6.47	9.57	11.36	11.27	16.64	24.69
CNCH6	3.30	6.25	9.46	10.99	10.68	16.09	28.19
CNCH7	3.17	6.50	9.65	11.20	10.83	16.42	26.52
FS1	4.67	8.32	11.59	13.39	13.16	19.02	27.05
FS2	2.76	3.77	4.00	4.25	4.54	7.21	12.72
FS3	4.68	6.60	8.95	10.06	11.55	20.22	33.53
FH1	3.31	6.29	9.67	12.12	14.09	17.05	22.87
FH3	4.08	6.65	9.83	11.08	10.85	15.96	22.93
FH4	3.29	6.45	9.67	11.31	10.77	16.36	26.34
Oak1	3.79	6.67	9.32	10.83	10.45	16.66	27.22
Oak2	4.31	6.55	9.20	10.71	10.17	16.26	23.87
Oak3	4.22	7.24	9.88	11.51	10.69	16.05	25.07
Oak4	4.60	7.23	9.85	11.48	10.26	14.37	24.08
Oak5	4.28	6.97	9.53	11.15	10.33	15.17	23.91
Oak6	3.94	6.75	9.29	10.94	10.23	15.58	24.43
Fir1	3.41	6.46	9.34	10.68	11.21	15.62	21.90
Fir2	3.56	6.58	9.39	10.68	11.09	15.75	21.92
Fir3	3.77	6.71	9.67	10.88	11.52	16.05	22.42
Fir4	3.78	6.66	9.57	10.79	11.52	15.91	22.14
Sound wood	3	7	9	10	12	18	30

In general, the EMC values obtained for blocks belonging to the same archaeological source but treated with different consolidants varied slightly and not significantly. At 100% RH, the CNCs treated blocks had EMC values slightly lower than wood treated with the other two consolidants and comparable with freeze-dried controls. At the same RH, the LNPs and BC treated blocks showed an EMC in average higher with respect to lyophilized controls (2–3 and 3–8% respectively). However, the observed differences were too low to be considered significant. Based on the obtained results it could be concluded that the moisture equilibrium content of treated samples was not influenced by the hygroscopic features of nano-scale consolidants. Obvious deviations from data uniformity were observed for the blocks S2, both those treated with nanoparticles and the freeze-dried control, CNCH1 and CNCH2 whose EMC values were on average 2–15% lower with respect to the others. This discrepancy can be explained taking into account what already mentioned above with respect to fir and elm samples, the presence of sediments inside the wood must have influenced its relation with moisture.

It is interesting to note that in the RH range used for conservative purposes (35–85%), the average EMC values for all blocks never exceeded the 18% considered as the threshold for the risk of biological attacks.

## Conclusion

The aim of the present work was to test three nano-scale consolidants based on cellulose and lignin. The obtained results did not allow for discriminating significant differences regarding their efficacy but they laid a profound base for more focused tests in future.

The problems in color changing observed for LNPs and BC could be solved by modifying the impregnation baths. For BC, a pigment compatible with the treated material could be added to the bath. For LNPs a careful selection of the wood source for lignin isolation could give LNPs of different color grades matching the color of the consolidation object (Zikeli et al., 2019).

Regarding the different penetration of the tested consolidants, it was not possible to identify relations neither with the state of preservation of wood nor with the shape and dimension of the nanoparticles. Thus, the different behavior must be related to chemical interactions between the nanoparticles themselves and between them and the wood. LNPs and CNCs penetration problems could be solved by modifying the impregnation conditions in the first case to facilitate penetration, in the second to prevent the gel formation. For BC a satisfying penetration was observed but the consolidating effect was not substantial. A best result could be achieved by modifying the shape of the nanocellulose or combining filamentous with spherical nanoparticles in order to obtain web-like structures more suitable to fill the voids and to support the cell wall, exploiting the potential of nano-scale consolidants to enter wood ultrastructure.

## Data Availability Statement

All datasets generated for this study are included in the article/Supplementary Material.

## Author Contributions

FA, GG, and MR conceived and planned the experiments. FZ and RN produced and provided the nanoparticles used in the experimentation. GS, FZ, and RN contributed to define the settings of the tests. MR and BP helped supervise the project. FA and GG wrote the paper with inputs from all authors. All the authors contributed to the discussion.

### Conflict of Interest

RN is employed by the company BioFaber srl. The remaining authors declare that the research was conducted in the absence of any commercial or financial relationships that could be construed as a potential conflict of interest.
