# Supplementary material for: Cellulose and Lignin Nano-Scale Consolidants for Waterlogged Archaeological Wood
Source: Front Chem. 2020 Jan 29;8:32. doi: 10.3389/fchem.2020.00032 (PMC7000621; doi:10.3389/fchem.2020.00032)
Supplement: Supplementary file 1 [file Table_1.DOCX]

Supplementary Material

# Supplementary Figures and Tables

## Supplementary Figures

**Supplementary Figure 1.** LNPs (A) and BC (B) treated blocks at the end of the consolidation treatment, a layer of nanoparticles is present on wood surface; (C) CNCs impregnation bath turned into a gel.

**Supplementary Figure 2.** Micro-morphological characterization of microbial decay. (A) Sample FTdis4 – silver fir, radial section stained with methylene blue: signs of degradation produced by erosion bacteria are well visible on the cell walls; (B) Sample FTdis4 – silver fir, radial section: fungal spores present inside the wood cells; (C-D) Sample FTdis9 – elm, cross section observed in bright-field (C) and polarized light (D): a severe loss of cellulose birefringence highlights that the crystalline structure of the polymer was almost completely lost due to the microbial attack Scale bars 20 µm.

**Supplementary Figure 3.** Aspect of the waterlogged blocks before the beginning of the consolidation treatment.

**Supplementary Figure 4.** Aspect of the freeze-dried controls, air-dried controls and treated blocks after the consolidation treatment and lyophilization. Wood equlibrated at 20 °C, 50% RH.

## Supplementary Tables

Tab. S1 Details of the tests performed to obtain stable BC suspensions

| **Test name** | **Procedure*** |
| --- | --- |
| T1 | - 0.5% Tween 20 + 1% Cinnamon oil + deionised water - Sonication 1h - 5% BC - Sonication 1h |
| T2 | - 0.5% Tween 20 + 1% Cinnamon oil + deionised water - Sonication 1h - 5% BC - Magnetic shaking 1h |
| T3 | - 0.5% Tween 20 + 1% Cinnamon oil + deionised water + 5% BC - Sonication 1h |
| T4 | - 0.5% Tween 20 + 1% Cinnamon oil + deionised water + 5% BC - Magnetic shaking 1h |
| T5 | - 0.5% Tween 20 + 1% Cinnamon oil + deionised water - Magnetic shaking 30 min - 5% BC - Magnetic shaking 1h |
| T6 | - 0.5% Tween 20 + deionised water - Sonication 1h - 5% BC - Magnetic shaking 1h |
| T7 | - 1% Tween 20 + deionised water - Sonication 1h - 5% BC - Magnetic shaking 1h |
| T8 | - 1% Tween 20 1% Cinnamon oil + deionised water - Sonication 1h - 5% BC - Magnetic shaking 1h |
| P1 | - 1% PEG 400 + 5% BC + deionised water - Sonication 1h - Magnetic shaking 1h |
| P2 | - 1% PEG 400 + 5% BC + deionised water - Magnetic shaking 1h |
| Z1 | - 1% sucrose + deionised water + 5% BC - Sonication 1h |
| Z2 | - 1% sucrose + deionised water + 5% BC - Magnetic shaking 1h |
| Z3 | - 1% sucrose + 5% BC + deionised water - Sonication 1h |
| Z4 | - 1% sucrose + 5% BC + deionised water - Magnetic shaking 1h |

*for all the tests sonicator amplitude was set at 50% and magnetic shaker speed at 500/600 rpm
