# Supplementary figures and images for: Cellulose and Lignin Nano-Scale Consolidants for Waterlogged Archaeological Wood
Source: Front Chem. 2020 Jan 29;8:32. doi: 10.3389/fchem.2020.00032 (PMC7000621; doi:10.3389/fchem.2020.00032)

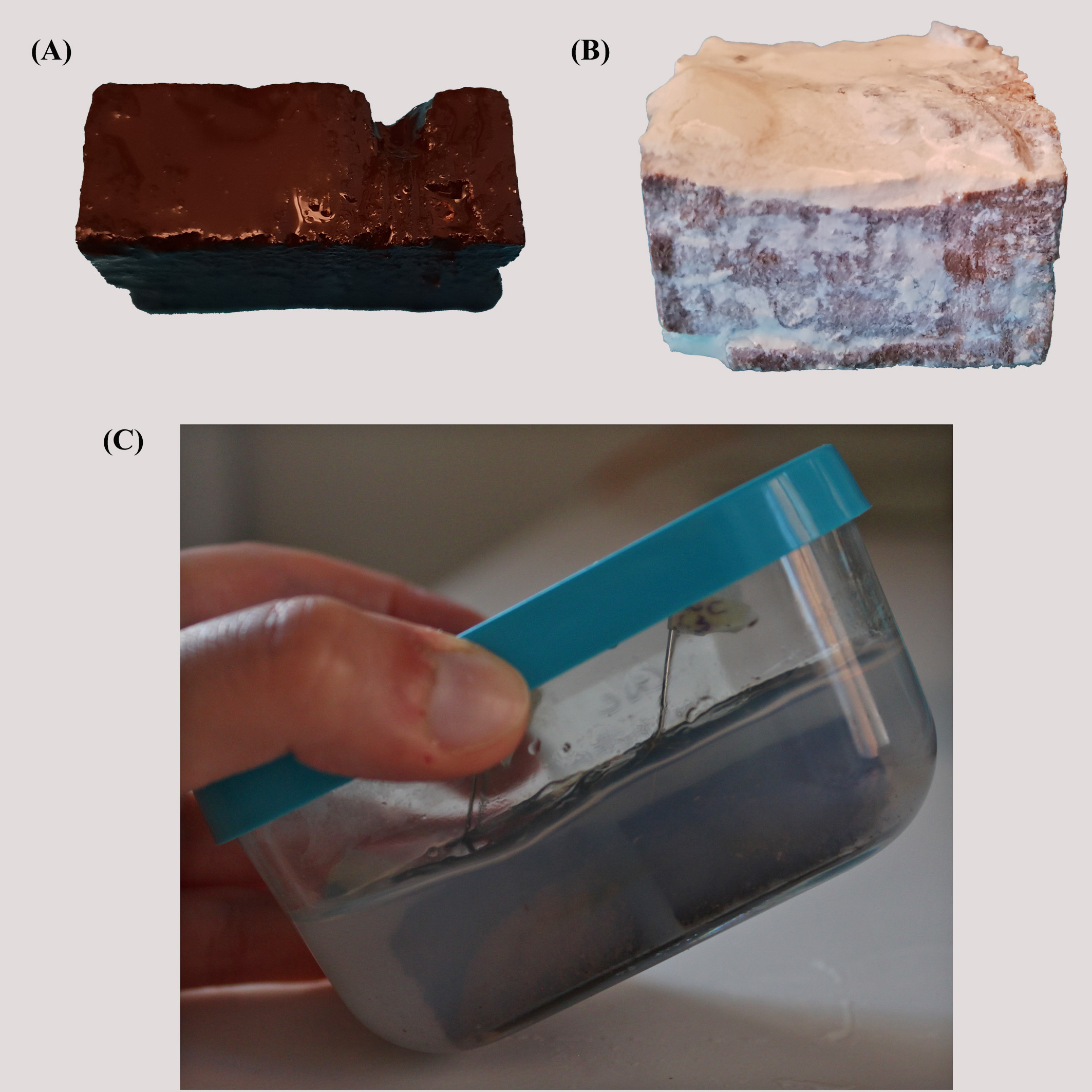

Supplement: Supplementary file 2 [file Image_1.JPEG]

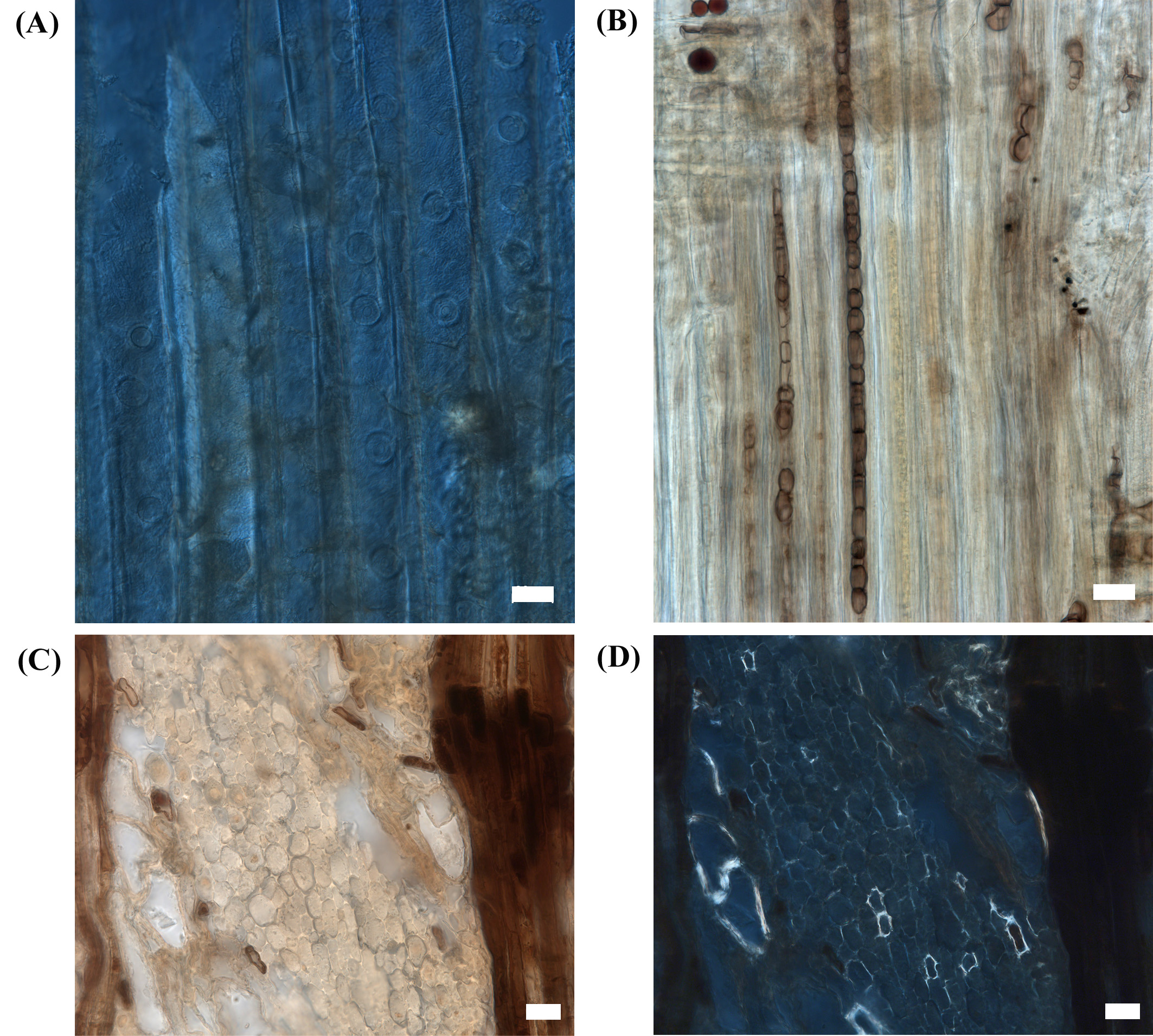

Supplement: Supplementary file 3 [file Image_2.JPEG]

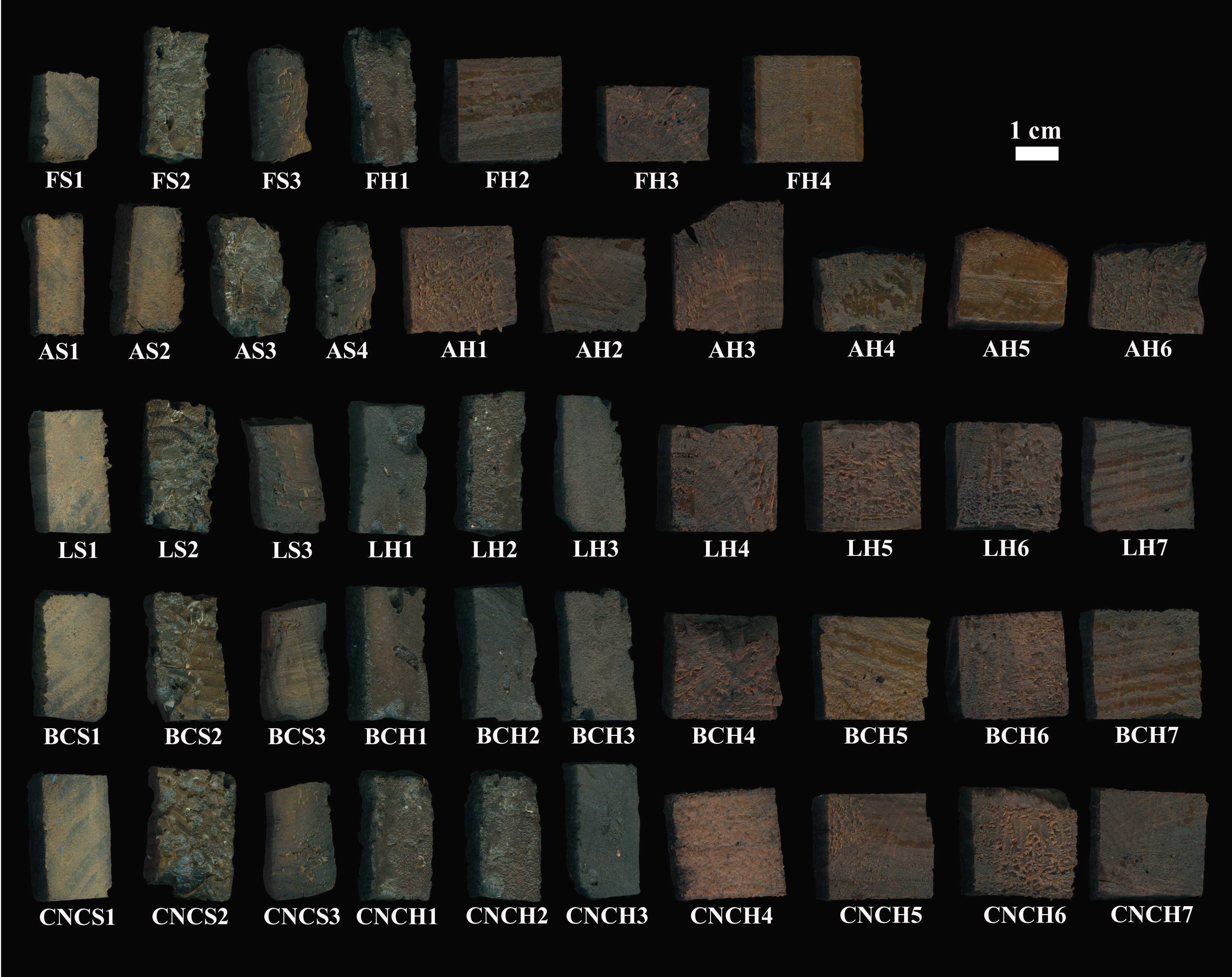

Supplement: Supplementary file 4 [file Image_3.JPEG]

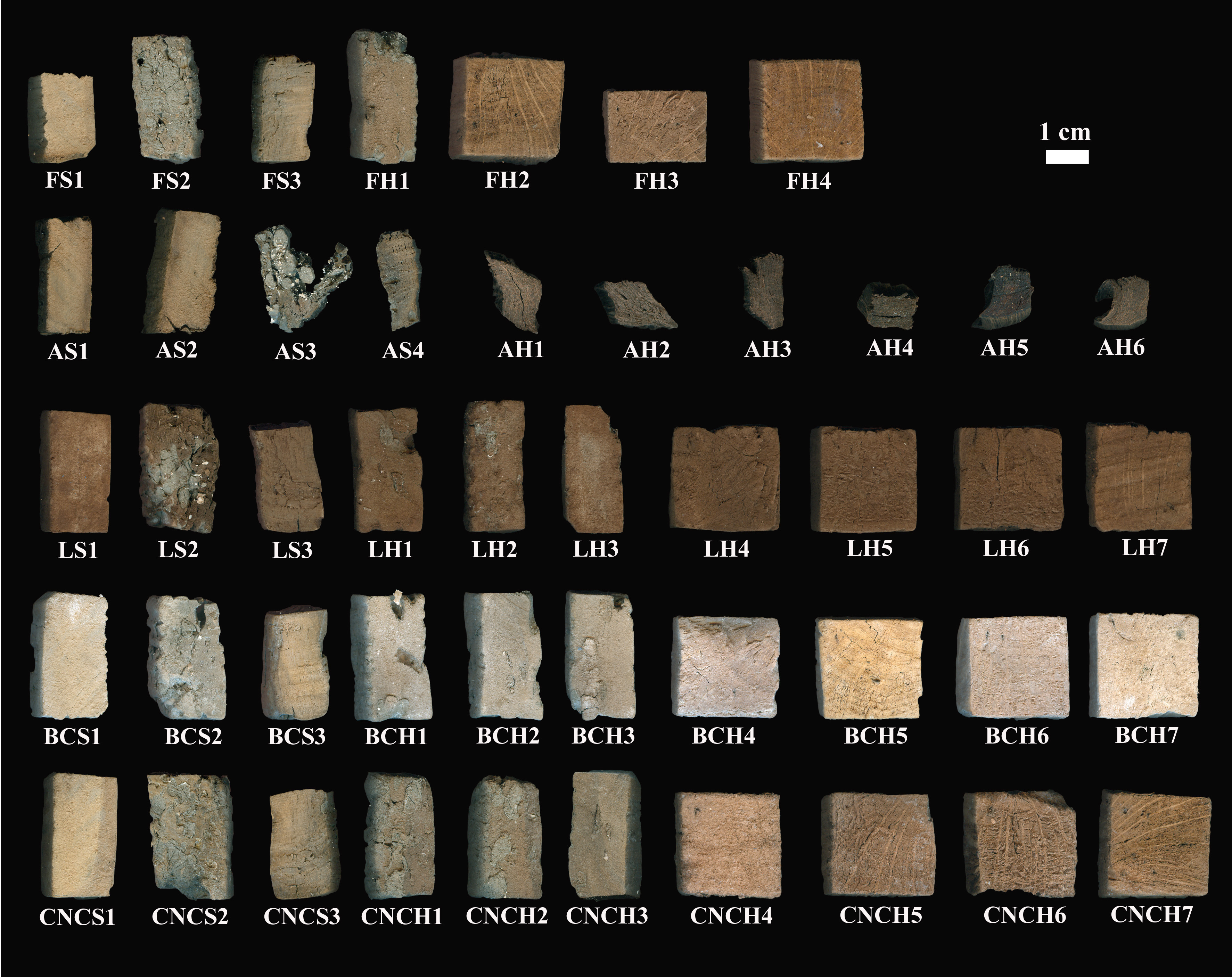

Supplement: Supplementary file 5 [file Image_4.JPEG]
